# Cognitive activation and school belonging: the mediating roles of emotional security and social inclusion in East Asia

**DOI:** 10.3389/fpsyg.2026.1795875

**Published:** 2026-05-18

**Authors:** Zhemin Zhu, Fengshu Liu, Bing Jia

**Affiliations:** 1 School of Educational Science, Beihua University, Jilin, China; 2 Department of Education, Faculty of Educational Sciences, University of Oslo, Oslo, Norway

**Keywords:** cognitively activating instruction, emotional security, PISA 2018, school belonging, social inclusion

## Abstract

**Introduction:**

This study examined how cognitively activating instruction is associated with students’ sense of school belonging through the psychological mechanisms of emotional security and social inclusion in East Asian educational contexts.

**Methods:**

Using PISA 2018 data from 40,330 students across six East Asian education systems, we estimated a parallel mediation model within a structural equation modeling framework while accounting for sampling weights and school-level clustering. Multi-group analyses were conducted to examine cross-system differences.

**Results:**

Cognitively activating instruction was positively associated with school belonging through both emotional security and social inclusion. The indirect effect via emotional security was stronger than that via social inclusion, while the direct effect remained significant, indicating partial mediation. Multi-group analyses revealed significant cross-system variation in the structural pathways, with the social inclusion pathway showing greater instability across education systems.

**Discussion:**

The findings suggest that cognitively activating instruction promotes school belonging through distinct socio-emotional mechanisms that are context-dependent. Emotional security appears to represent a more consistent pathway across East Asian education systems than social inclusion.

## Introduction

Periods of heightened global uncertainty, characterized by geopolitical tensions, institutional realignment, and rapid social change, have renewed scholarly attention to the foundations of social cohesion and social inclusion. Increasingly, research suggests that social cohesion depends not only on political and economic arrangements, but also on everyday institutional contexts in which individuals experience recognition, participation, and belonging. Schools are central to this process, as they function both as sites of academic learning and as key environments for socialization during adolescence.

Schools are expected to promote cognitive development through effective instruction while also fostering students’ sense of belonging. A substantial body of research has shown that students’ sense of school belonging is associated with academic engagement, motivation, and psychological well-being, and is inversely related to maladjustment and dropout risk (Goodenow and Grady, 1993; Allen et al., 2018). Despite its importance, however, the classroom-level processes through which instructional practices shape students’ sense of belonging remain insufficiently understood, particularly in large-scale and cross-national research. One instructional dimension that has received growing attention is cognitively activating instruction. This form of instruction emphasizes higher-order thinking, conceptual understanding, and engagement with challenging tasks. Research on teaching quality has consistently demonstrated that cognitively activating classroom practices are associated with students’ learning gains and deeper understanding (Seidel and Shavelson, 2007). Much less is known about how such instructional practices affect students’ psychological experiences and social inclusion at school. From a psychological perspective, cognitively demanding instruction may influence students’ sense of school belonging through multiple pathways. It has been shown to be associated with reduced well-being and maladaptive outcomes when instructional demands exceed students’ perceived control or coping resources (Pekrun et al., 2002; Pekrun, 2006). At the same time, instructional practices may shape students’ social experiences in the classroom. Perceived social exclusion poses a direct threat to the fundamental need to belong (Baumeister and Leary, 2007).

Although emotional security and social inclusion are conceptually related, they represent different psychological processes. Emotional security concerns whether students experience schoolwork and evaluation as manageable, predictable, and non-threatening. It reflects students’ internal sense of safety in relation to academic demands. Social inclusion, by contrast, concerns students’ perceived acceptance, recognition, and relational embeddedness within the peer environment. It reflects whether students feel that they are socially integrated rather than marginalized at school. Both processes are theoretically relevant to school belonging, because belonging is shaped not only by students’ emotional experience of school as a safe environment, but also by their perception of being accepted and included in the school community. Cognitively activating instruction may therefore be linked to school belonging through two parallel pathways: an emotional pathway related to reduced threat and uncertainty (emotional security), and a social pathway related to participation and peer connectedness (social inclusion).

Emotional security and social inclusion are both theoretically relevant, but they are rarely examined simultaneously as distinct psychological mechanisms linking instruction to school belonging. In addition, comparative research indicates substantial cross-national variation in students’ emotional well-being and social experiences at school (OECD, 2017), suggesting that instructional practices may carry different psychological meanings across contexts. Against this background, the present study examines how cognitively activating instruction is associated with students’ sense of school belonging through parallel pathways involving school-related emotional security and perceived social inclusion, using large-scale data from multiple East Asian education systems. We propose the following hypotheses:

*H1*: Cognitively activating instruction is positively associated with students’ emotional security in school.

*H2*: Cognitively activating instruction is positively associated with students’ social inclusion in school.

*H3*: Emotional security is positively associated with students’ sense of school belonging.

*H4*: Social inclusion is positively associated with students’ sense of school belonging.

*H5*: Emotional security and social inclusion mediate the association between cognitively activating instruction and school belonging.

Using data from PISA 2018, this study applies structural equation modeling with multigroup analyses to examine the relationships among instructional practices, students’ psychological experiences, and their sense of school belonging across East Asian education systems. The analysis draws on student questionnaire data and combines weighted pooled SEM with country/region-level multi-group SEM to assess both overall patterns and cross-system variation. The pooled models examine whether Emotional security and social inclusion function as parallel psychological pathways linking cognitive activation to school belonging, while country/region-specific analyses evaluate the robustness and contextual sensitivity of these pathways. Rather than focusing on cross-country/region associations as descriptive comparisons, the study mainly focuses on identifying a classroom-based psychological mechanism through which instructional practices shape students’ emotional security and social inclusion.

## Program for international student assessment

The Programme for International Student Assessment (PISA) is widely regarded as a highly influential global educational assessment. It evaluates the literacy of 15-year-old students in the core domains of reading, mathematics, and science. PISA has significantly impacted educational policy and practice globally by broadening the scope of international comparison, enhancing the interpretation of results, and guiding national educational decisions (Breakspear, 2012; OECD, 2013). Administered every 3 years, the most recent complete cycle with publicly available microdata suitable for secondary analysis is PISA 2018. The primary focus of PISA 2018 was reading literacy, with secondary assessments in mathematics and science. Although PISA 2022 provides extensive background questionnaires and scaled scores, detailed item-level data for mathematics from recent cycles are not publicly released.

Research utilizing PISA data to analyze cross-national educational traits has become a vital method for comprehending systemic variations and guiding international educational enhancement. Scholarship based on PISA can be categorized into four principal areas. One area investigates determinants linked to student achievement and literacy outcomes. This includes studies on gender disparities in performance (Liu et al., 2008), the impact of information and communication technology on learning (Zhang and Liu, 2016), and cross-national examinations of learning progressions (Zhu, 2023; Jia and Zhu, 2025). Another area concentrates on the psychology of learning, exploring student affect and motivation. This body of work addresses learning anxiety (Foley et al., 2017; Luttenberger et al., 2018), academic self-concept and self-efficacy (Lee, 2009), and learning motivation (Oden, 2020), underscoring the relevance of internal psychological processes for explaining international differences in outcomes. A third area is dedicated to psychometric and item-level evaluation of the PISA instrument itself. This includes methodological investigations using PISA data and analyses of mathematical literacy via specific test items, primarily contributing to advancements in measurement and the interpretation of assessment data.

A fourth and more recent research direction focuses on the influence of the school environment on student wellbeing. Evidence from PISA indicates significant concerns regarding adolescent emotional and social health. Only approximately two thirds of students report general life satisfaction (OECD, 2019). These statistics highlight that the school experience encompasses more than academic results and is deeply connected to psychological state. Research confirms that cognitive, psychological, and social well-being metrics coalesce into a unified welfare construct in education, with student resilience, fear of failure, and sense of belonging serving as its core elements (Govorova et al., 2020). Within PISA, resilience is often measured as self-efficacy in adapting to challenges. Studies note the significance of teacher well-being, proposing that supportive educators contribute to positive student outcomes, encapsulated by the idea that teacher satisfaction promotes student happiness (Edwards, 2020). Additional findings suggest that relational factors shape student aspirations; for instance, while positive student-teacher relationships and school belonging both foster educational expectations, only school belonging correlates with higher career expectations (Wong et al., 2019). Concurrently, national PISA performance is closely tied to cognitive ability and socioeconomic factors like national income, illustrating the structural context linking achievement and well-being (Boman, 2023). Recent work also indicates that constructive teacher feedback enhances students’ school belonging, partially mediated by their subjective well-being (Li et al., 2024).

Prior research has often considered instructional methods, psychological well-being, and social inclusion as separate or weakly associated concepts, without thoroughly modeling the causal pathway from classroom instruction to social outcomes. In educational research, cognitive activation is recognized as a crucial aspect of teaching quality, involving practices that stimulate higher-order thinking and problem-solving (Klieme et al., 2009). While its academic benefits are documented, its effects on student psychological and social development are not fully explained. There is a scarcity of cross-country/region studies that employ group-specific SEM analyses to investigate this mechanism. Specifically, the process through which cognitively activating instruction affects students’ psychological experiences, and how this subsequently influences school belonging, remains inadequately examined in cross-system SEM research.

## Materials and methods

### Data source and sample

The present analyses focus on a subset of East Asian education systems, including Hong Kong, Japan, Korea, Macao, Chinese mainland, and Chinese Taipei. These systems were selected due to their shared regional context and their substantial variation in instructional practices and student experiences. The analytic sample was restricted to students with valid information on key instructional, psychological, and outcome variables. Student sampling weights provided by PISA were applied in all analyses to ensure population-representative estimates. Given the nested structure of the data, with students clustered within schools, school identifiers were used to account for non-independence of observations.

Cognitive activation and school belonging were measured using PISA-provided IRT indices (STIMREAD and BELONG). Emotional security and social inclusion were modeled as latent variables, indicated by six anxiety items (ST038Q03NA – ST038Q08NA) and three social exclusion items (ST183Q01HA – ST183Q03HA), respectively. These items are scored using reverse scoring. Socioeconomic status, gender, immigrant background, and grade repetition were included as covariates. All the dimensions are shown in Table 1.

**Table 1:** Measures, sample items, and scaling.

**Dimension**	**Item code(s)**	**Sample item description**
Cognitive activation	STIMREAD	Frequency of instructional practices that require explanation, discussion of ideas, engagement with cognitively demanding tasks, and learning from errors
Social inclusion (low perceived social exclusion)	ST183Q01HA	Feeling left out at school
	ST183Q02HA	Feeling isolated from peers at school
	ST183Q03HA	Feeling like an outsider at school
Emotional security (low school-related anxiety)	ST038Q03NA	Feeling nervous or anxious about schoolwork
	ST038Q04NA	Worrying about poor academic performance
	ST038Q05NA	Feeling stressed when studying or doing school tasks
	ST038Q06NA	Feeling tense when facing difficult schoolwork
	ST038Q07NA	Worrying about making mistakes in school
	ST038Q08NA	Feeling anxious in evaluative school situations
School belonging	BELONG	Feeling accepted, included, and attached to the school community
Socioeconomic status	SES	Economic, social, and cultural status based on family background
Gender	ST004D01T	Student gender
Immigrant background	IMMIG	Immigrant status of the student
Grade repetition	REPEAT	Whether the student has repeated a grade

Anxiety items (ST038Q03NA–ST038Q08NA) and social exclusion items (ST183Q01HA–ST183Q03HA) were reverse-coded prior to analysis, such that higher scores indicate lower school-related anxiety and lower perceived social exclusion, respectively. Accordingly, the latent constructs are interpreted as emotional security and social inclusion in the structural models. Cognitive activation, school belonging, and socioeconomic status were measured using PISA-provided IRT indices.

Measures, sample items, and scaling information for all study variables are summarized in Table 1. Cognitive activation, school belonging, and socioeconomic status were measured using PISA-provided IRT indices, while emotional security and social inclusion were modeled as latent constructs indicated by multiple student questionnaire items.

The sample size is shown in Table 2.

**Table 2:** Sample size by education system (PISA 2018).

Education system	Sample size (*N*)
Hong Kong (China)	5,511
Japan	5,818
Korea	6,494
Macao (China)	3,715
Chinese mainland (four provinces/municipalities)	11,830
Chinese Taipei (China)	6,962
Total	40,330

Sample sizes refer to students with valid data on the focal instructional, psychological, and outcome variables.

Table 2 shows that the final analytical sample comprised 40,330 students from six East Asian education systems participating in PISA 2018. Sample sizes varied across systems, ranging from 3,715 students in Macao (China) to 11,830 students in Chinese mainland (four provinces/municipalities). The remaining systems included Hong Kong (China) (*N* = 5,511), Japan (*N* = 5,818), Korea (*N* = 6,494), and Chinese Taipei (China) (*N* = 6,962). All analyses incorporated PISA student sampling weights to ensure population-representative estimates despite differences in national sample sizes.

### Cognitive activation

Cognitive activation was measured using the PISA 2018 index of cognitively activating instruction (STIMREAD). This index reflects students’ perceptions of instructional practices that emphasize higher-order thinking and active engagement, such as explaining one’s reasoning, discussing alternative solutions, engaging with cognitively demanding tasks, and learning from mistakes. The index was constructed by the OECD using item response theory (IRT) scaling procedures, with higher values indicating more frequent exposure to cognitively activating instruction.

### Psychological processes

Two psychological constructs were examined as mediators and were modeled as latent variables.

Emotional security (low school-related anxiety) was measured using six items from the PISA 2018 student questionnaire (ST038Q03NA–ST038Q08NA). This interpretation is grounded in the content of the items themselves, which refer specifically to students’ nervousness, stress, tension, worry about mistakes, and anxiety in evaluative school situations. Conceptually, such feelings reflect not only negative affect, but also students’ perceptions of threat, uncertainty, and limited control in academic contexts. From the perspective of control-value theory, school-related anxiety arises when students attach importance to academic demands but feel insufficient control over performance and evaluation. Accordingly, lower endorsement of these items indicates more than the mere absence of anxious affect; it suggests that students experience schoolwork and evaluation as more manageable, less threatening, and more psychologically safe. For this reason, the reverse-coded latent factor is interpreted as emotional security in school rather than mere reduced anxiety.

Social inclusion was operationalized using three reverse-coded items on perceived social exclusion at school (ST183Q01HA–ST183Q03HA). The original items refer to feeling left out, isolated, or like an outsider at school, and thus capture students’ perceived relational marginalization within the school community. Conceptually, these items concern students’ perceived position in the peer environment and whether they experience themselves as socially accepted and relationally embedded rather than excluded. Accordingly, higher scores on the reverse-coded latent factor were interpreted as representing stronger social inclusion. Thus, we interpret the reverse-coded factor not merely as reduced social exclusion, but as an indicator of social inclusion in school.

Taken together, these two constructs capture distinct but complementary pathways through which classroom instruction may shape school belonging. Emotional security reflects students’ internal experience of academic safety, whereas social inclusion reflects their perceived relational position within the school community. Modeling them simultaneously therefore allows the present study to examine whether cognitively activating instruction is associated with school belonging through emotional, social, or overlapping psychological processes. Modeling emotional security and social inclusion as latent constructs allowed measurement error to be explicitly accounted for in the structural equation models.

### School belonging

Students’ sense of school belonging was measured using the PISA 2018 index of school belonging (BELONG). This index reflects students’ feelings of acceptance, inclusion, and attachment to their school community. It was constructed using IRT-based scaling procedures by the OECD, with higher values indicating a stronger sense of school belonging.

### Covariates

Several student-level background variables were included as covariates to reduce potential confounding effects, including socioeconomic status (SES), gender, immigrant background, and grade repetition. All covariates were treated as observed variables and were allowed to predict cognitive activation, the psychological mediators, and school belonging in the structural models.

### Analytical strategy

Structural equation modeling (SEM) was employed to examine the hypothesized pathways linking cognitively activating instruction to students’ sense of school belonging. Emotional security and social inclusion were specified as parallel mediators, and their residual covariance was freely estimated to account for shared psychological variance not explained by instructional practices. A direct path from cognitive activation to school belonging was included for testing partial mediation.

To reduce potential omitted-variable bias, background variables (socioeconomic status, gender, immigrant background, and grade repetition) were included as predictors of cognitive activation, the two mediators, and school belonging. All models were estimated using robust maximum likelihood (MLR) with full information maximum likelihood to handle missing data. Student sampling weights were incorporated, and standard errors were adjusted for school-level clustering. To examine cross-system differences, multi-group SEM was conducted across six East Asian education systems. Differences in structural paths were tested by comparing a freely estimated model with a model in which paths were controlled to be equal across groups using chi-square difference tests. Indirect effects were estimated using bootstrap procedures, and 95% confidence intervals were reported.

### Model evaluation

Model fit was evaluated using multiple indices, including the comparative fit index (CFI), Tucker–Lewis index (TLI), root mean square error of approximation (RMSEA), and standardized root mean square residual (SRMR). Conventional cutoff values were used to assess acceptable model fit. Statistical significance was evaluated using two-tailed tests with an alpha level of 0.05.

### Software

All analyses were conducted in R using the lavaan package for structural equation modeling. Model estimation accounted for robust standard errors, student sampling weights, and school-level clustering in accordance with OECD recommendations for PISA data analysis.

### Model

**Figure 1:**
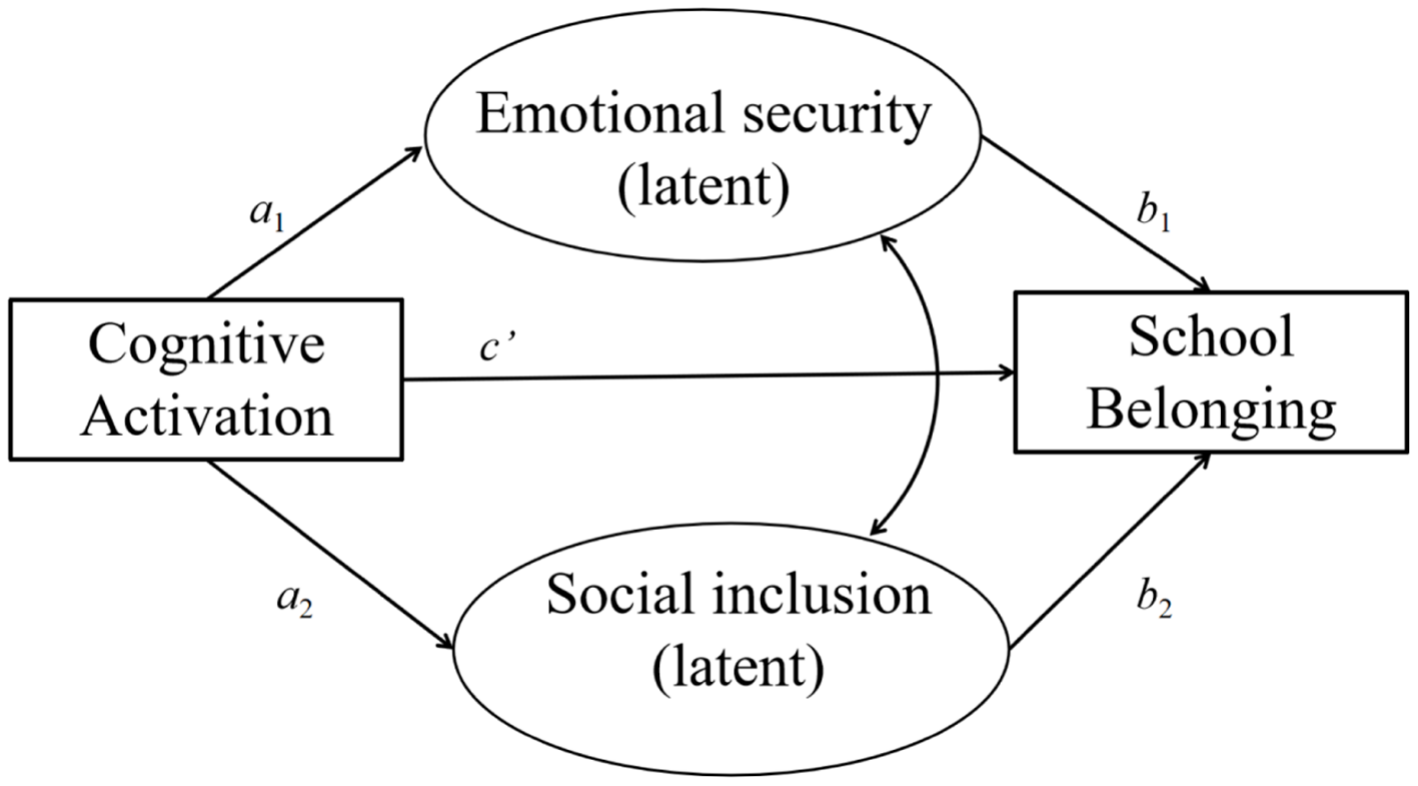
The hypothetical mediation model. Emotional security and social inclusion are allowed to covary to account for shared psychological variance not explained by instructional practices. Student background variables (socioeconomic status, gender, immigrant background, and grade repetition) are included in the model but omitted from the figure for clarity.

The hypotheses outlined above jointly constitute the hypothetical mediation model shown in Figure 1.

Figure 1 illustrates the hypothesized mediation model examining the psychological pathways through which cognitively activating instruction is associated with students’ sense of school belonging. Cognitive activation is specified as the focal instructional predictor, with emotional security and social inclusion modeled as parallel psychological mediators. A direct path from cognitive activation to school belonging is included to test for partial mediation.

## Results

All five hypotheses were supported in the pooled model. Cognitively activating instruction was positively associated with both emotional security and social inclusion, and both mediators were positively related to school belonging. In addition, the indirect effects via emotional security and social inclusion were both statistically significant, supporting the proposed mediation model.

### Descriptive statistics and model fit

The pooled structural equation model demonstrated good overall fit to the data. Results are shown in Table 3. Model fit indices indicated that the hypothesized parallel mediation model adequately represented the observed covariance structure. Specifically, the comparative fit index was 0.960, the Tucker Lewis index was 0.942, the root mean square error of approximation was 0.043, and the standardized root mean square residual was 0.020. These values meet commonly accepted criteria for good model fit in structural equation modeling.

**Table 3:** Model fit indices for the pooled structural equation model.

χ^2^	df	CFI	TLI	RMSEA	SRMR
5255.56	68	0.96	0.942	0.043	0.02

### Measurement model results

The measurement models for emotional security and social inclusion demonstrated satisfactory psychometric properties. All factor loadings were statistically significant at the 0.001 level. Standardized factor loadings for the emotional security indicators ranged from 0.64 to 0.72, while standardized factor loadings for the social inclusion indicators ranged from 0.68 to 0.88, indicating adequate indicator coherence for both latent constructs.

The internal consistency of the two latent constructs was satisfactory. Cronbach’s alpha was 0.839 for emotional security and 0.817 for social inclusion. Composite reliability (CR), calculated based on standardized factor loadings, showed similar values (CR = 0.837 for emotional security; CR = 0.818 for social inclusion), indicating good reliability of both constructs.

### Structural model results for the pooled sample

**Figure 2:**
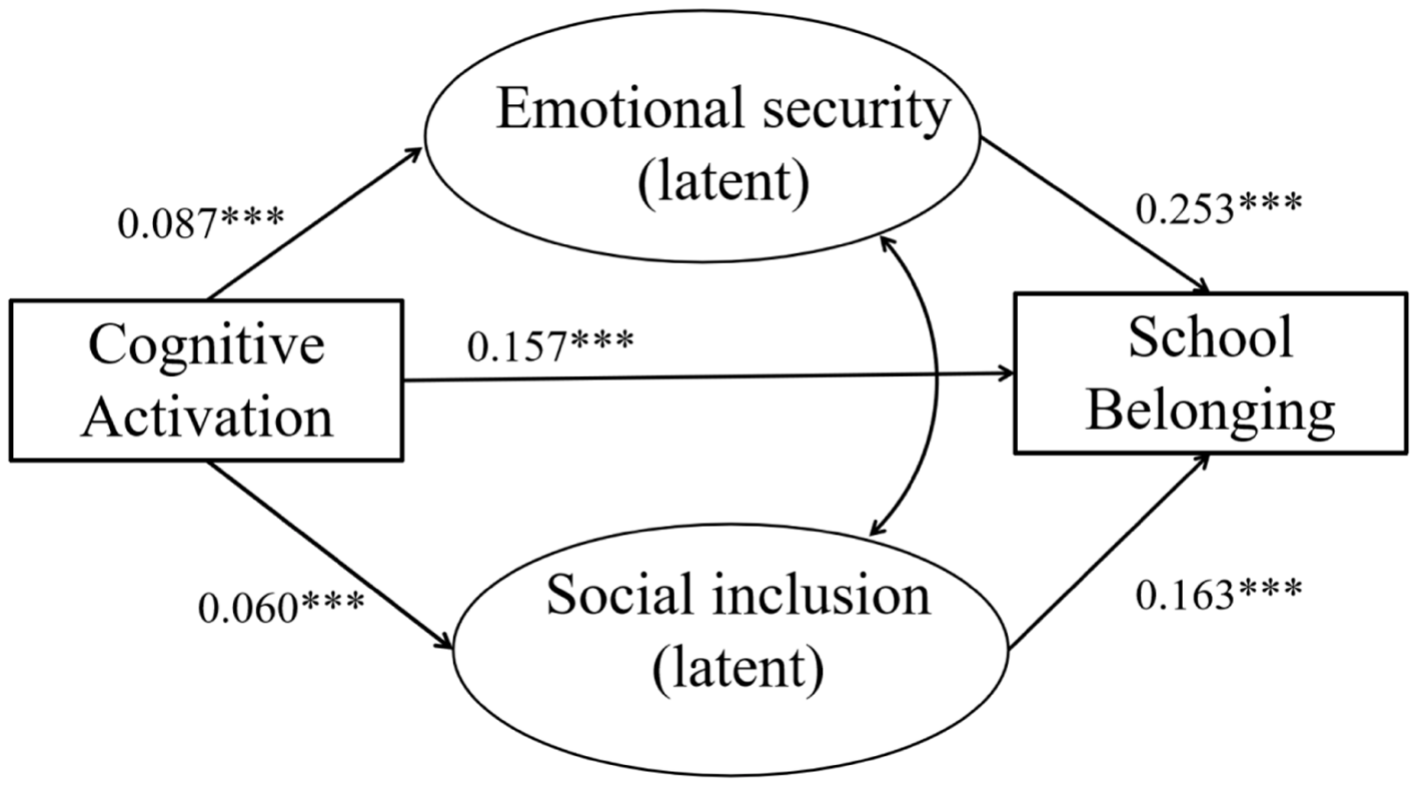
Standardized pooled structural equation model. Standardized coefficients (β). ***p < 0.001. All models account for student sampling weights and school-level clustering.

The standardized results of the pooled structural equation model are shown in Figure 2. Cognitive activation was positively associated with emotional security and social inclusion, indicating that higher levels of cognitively activating instruction were related to higher levels of emotional security and social inclusion. Both psychological mediators were strongly associated with school belonging. Higher levels of emotional security and stronger social inclusion were both associated with higher levels of school belonging. After accounting for the two mediators, the direct path from cognitive activation to school belonging remained statistically significant, indicating partial mediation.

Figure 2 presents the standardized results of the pooled structural equation model estimated across six East Asian education systems. The model shows both the direct association between cognitively activating instruction and school belonging and the indirect pathways operating through emotional security and social inclusion. Standardized path coefficients are displayed alongside each path. In the pooled model, cognitively activating instruction was positively associated with emotional security (*β* = 0.09, *p* < 0.001) and social inclusion (*β* = 0.06, *p* < 0.001). In turn, both emotional security (*β* = 0.25, *p* < 0.001) and social inclusion (*β* = 0.16, *p* < 0.001) were positively associated with school belonging. The direct effect of cognitive activation on school belonging also remained significant (*β* = 0.16, *p* < 0.001), indicating partial mediation.

To further examine the mediating mechanisms, standardized indirect effects were estimated using bootstrap procedures.

Table 4 shows that the indirect effect via emotional security was statistically significant. The indirect effect via social inclusion was also statistically significant, but smaller in magnitude. The total indirect effect was 0.029, indicating that both mediators jointly contributed to the association between cognitive activation and school belonging. Based on standardized estimates, the indirect effect via emotional security was approximately *β* = 0.022, whereas the indirect effect via social inclusion was approximately *β* = 0.010.

**Table 4:** Direct and indirect effects (unstandardized estimates).

Effect	Estimate	SE	*p*	95% CI
Direct effect	0.142	0.007	<0.001	[0.127, 0.156]
Indirect via emotional security	0.020	0.002	<0.001	[0.015, 0.025]
Indirect via social inclusion	0.009	0.002	<0.001	[0.006, 0.012]
Total indirect effect	0.029	0.003	<0.001	[0.023, 0.035]
Total effect	0.171	0.008	<0.001	[0.155, 0.186]

Overall, these findings suggest that both emotional security and social inclusion significantly mediate the relationship between cognitively activating instruction and school belonging, with emotional security representing the stronger pathway. The indirect effect via emotional security can be considered small, whereas the effect via social inclusion approaches a trivial magnitude. Despite their relatively modest size, these effects remain meaningful given the large sample and the complexity of the educational processes involved.

In addition to the main structural paths, the effects of control variables were examined. Overall, these effects were relatively small and did not alter the main relationships among cognitive activation, emotional security, social inclusion, and school belonging. These results are reported in Supplementary Table S1.

### Cross country/region variation in structural pathways

Cross-system variation in structural pathways was examined using multi-group structural equation modeling. Formal model comparison indicated significant cross-system differences in the structural paths, suggesting that the associations among cognitive activation, the two mediators, and school belonging were not fully invariant across education systems. Across all systems, emotional security was consistently and positively associated with school belonging, indicating a relatively robust psychological pathway linking cognitively activating instruction to belonging. By contrast, the association between cognitive activation and social inclusion varied more substantially across systems, suggesting that the social implications of cognitively activating instruction are more context-dependent. Country- or region-specific standardized path estimates are reported in Table 5.

**Table 5:** Country/region-specific standardized structural path estimates.

Path	Hong Kong (China)	Japan	Korea	Macao (China)	Chinese mainland (four provinces/ municipalities)	Chinese Taipei (China)
Cognitive activation → Emotional security	0.075***	0.043*	0.040*	0.106***	0.184***	0.052**
Cognitive activation → Social inclusion	-0.004 ns	−0.081***	0.129***	−0.018 ns	0.106***	−0.098***
Emotional security → School belonging	0.251***	0.228***	0.170***	0.275***	0.224***	0.231***
Social inclusion → School belonging	0.125***	0.163***	0.198***	0.150***	0.247***	0.068***
Cognitive activation → School belonging (direct)	0.193***	0.150***	0.193***	0.195***	0.214***	0.145***

Standardized coefficients are reported. ***p < 0.001, **p < 0.01, *p < 0.05, ns = not significant.

To formally test cross-system differences, a chi-square difference test was conducted by comparing a freely estimated multi-group model with a model in which structural paths were controlled to be equal across groups. The results indicated a significant deterioration in model fit when equality constraints were imposed (Δχ^2^ = 686.31, Δdf = 25, *p* < 0.001), suggesting that the structural relationships differ across educational systems.

Country/region-specific indirect effects further illustrated the heterogeneity of the psychological pathways linking cognitively activating instruction to school belonging. Across all six education systems, the indirect effect of cognitive activation on school belonging through emotional security was consistently positive, indicating a robust and common psychological pathway across East Asian contexts. In contrast, the indirect effect through social inclusion varied substantially in both magnitude and direction across education systems. This pathway was positive and relatively stronger in Chinese mainland and Korea, close to zero in Hong Kong and Macao, and negative in Japan and Chinese Taipei. These patterns suggest that the social implications of cognitively activating instruction are more sensitive to contextual and cultural conditions than its emotional implications. Taken together, the findings indicate that emotional security constitutes a more generalizable psychological mechanism linking cognitively activating instruction to school belonging, whereas social inclusion operates as a more context-dependent and less stable pathway across East Asian education systems.

## Discussion

This study investigated how cognitively activating instruction is associated with students’ sense of school belonging through distinct psychological pathways involving emotional security and social inclusion, using large-scale data from six East Asian education systems. By modeling these mechanisms simultaneously within a weighted structural equation framework, the study responds to calls for research that moves beyond achievement outcomes to examine how classroom instruction shapes students’ psychological experiences and social inclusion.

### Emotional security as a robust pathway linking instruction to belonging

A central finding is that cognitively activating instruction is consistently associated with higher emotional security, which in turn is strongly related to students’ sense of school belonging across all education systems examined. The indirect effect through emotional security was statistically significant in the pooled model and remained stable across national contexts, suggesting that reduced school-related anxiety constitutes a generalizable psychological mechanism linking instructional practices to belonging.

This finding extends prior research on cognitively activating instruction, which has primarily emphasized its role in promoting deeper learning and academic achievement (Seidel and Shavelson, 2007; Klieme et al., 2009), by demonstrating its relevance for students’ emotional experiences. Although cognitively demanding instruction has sometimes been linked to heightened anxiety when instructional demands are perceived as excessive or evaluative (Pekrun et al., 2002; Pekrun, 2006), the present results suggest a more nuanced interpretation. Instructional practices that emphasize explanation, conceptual understanding, and learning from errors may reduce uncertainty and evaluative threat, thereby enhancing students’ emotional security. In line with control-value theory, such emotionally supportive learning environments are more likely to foster adaptive engagement and positive self-related outcomes, including a stronger sense of belonging.

The consistency of this pathway across diverse East Asian systems is particularly noteworthy given documented cross-national differences in students’ emotional well-being (OECD, 2017). It suggests that emotional security represents a foundational psychological condition through which instructional practices influence students’ attachment to school, regardless of broader cultural or institutional differences.

### Social inclusion as a context-dependent mechanism

Compared with emotional security, social inclusion showed a weaker and more variable mediating role across education systems. In the pooled model, the indirect effect through social inclusion was statistically significant but small, suggesting that cognitively activating instruction may support school belonging through peer-related processes, although less consistently than through emotional security. While social inclusion was positively associated with school belonging in all contexts, its linkage to cognitively activating instruction differed markedly by country/region. This pattern suggests that the social consequences of cognitively activating instruction are more context-dependent. In some systems, such instruction may promote peer discussion and mutual recognition, whereas in others it may be experienced as more performance-oriented, limiting its contribution to inclusive peer dynamics.

### Partial mediation

The persistence of a significant direct effect suggests that the relationship between cognitive activation and school belonging is only partially mediated by emotional security and social inclusion. This indicates that additional mechanisms may be at work.

One possible explanation can be drawn from Social Identity Theory. Cognitively activating instruction often involves communicating high expectations, providing challenging tasks, and engaging students in meaningful academic work. Such practices may signal to students that they are valued members of the classroom community, thereby strengthening their identification with the group and enhancing their sense of belonging.

Another perspective is offered by Relational Frame Theory. Cognitively demanding classroom interactions may foster perspective-taking and relational understanding, enabling students to better situate themselves within the social and academic environment. Through these processes, students may develop a stronger sense of connectedness that is not fully captured by measures of emotional security or social inclusion.

These interpretations suggest that cognitively activating instruction may influence school belonging through both emotional–social pathways and more direct identity- and cognition-based mechanisms. Future research could further examine these processes by incorporating measures of perceived recognition, identity formation, or perspective-taking to better disentangle the multiple pathways linking instructional practices to student outcomes.

### Differences across the six east Asian education systems

Differences in high-stakes examination systems may shape how cognitively activating instruction relates to students’ social experiences. In Chinese mainland and Korea, highly competitive and standardized entrance examinations dominate lower secondary education. Under such conditions, classroom practices are strongly aligned with performance and ranking. In these contexts, cognitively activating instruction may be more likely to foster shared academic engagement and peer recognition, thereby strengthening social inclusion. In contrast, Japan and Chinese Taipei adopt more diversified admission systems that incorporate non-academic indicators. However, these systems may also heighten evaluative pressure and peer comparison within classrooms, which could weaken or even reverse the link between cognitively activating instruction and students’ perceived social inclusion.

Cultural norms regarding authority and evaluation may also contribute to these differences. In China and Korea, influenced by traditional Confucianism, teachers hold a higher status than those in Japan and Chinese Taipei. In GTSI 2018, China ranked first, South Korea second, and Japan eleventh (Dolton et al., 2018). In contexts where teacher authority is more strongly emphasized, students may perceive evaluation as the exclusive domain of teachers, limiting the salience of peer-related processes. By contrast, in systems more influenced by student-centered or participatory educational philosophies, students may be more receptive to multiple perspectives in learning, making social inclusion a more relevant mechanism. The weak or insignificant association between cognitive activation and social inclusion observed in Hong Kong and Macao may be related to their unique education systems. These interpretations remain tentative but suggest that the social implications of instructional practices may depend on how competition, evaluation, and peer interaction are organized within specific educational systems.

### Regional similarities and differences in China

Within the four Chinese societies examined—Hong Kong, Macao, Chinese mainland, and Chinese Taipei—both common patterns and notable differences were observed. A clear point of convergence was the emotional security pathway. In all four contexts, cognitively activating instruction was positively associated with emotional security, and emotional security was in turn a strong positive predictor of school belonging. This pattern suggests that emotional security constitutes a relatively robust and shared mechanism linking cognitively activating instruction to school belonging across Chinese societies.

By contrast, the social inclusion pathway showed greater regional divergence. In Chinese mainland, cognitively activating instruction was positively associated with social inclusion, and social inclusion was also strongly related to school belonging, indicating a relatively strong social pathway. In Chinese Taipei, however, the path from cognitively activating instruction to social inclusion was negative, although social inclusion itself remained positively associated with school belonging. In Hong Kong and Macao, the same path was non-significant, even though social inclusion continued to predict school belonging positively. Taken together, these findings suggest that while emotional security represents a broadly shared pathway across Chinese societies, the social inclusion pathway is more differentiated and context-sensitive, varying substantially in both magnitude and direction.

### Implications and limitations

Together, these findings underscore the importance of integrating instructional quality, psychological processes, and social outcomes within a unified analytical framework. For educational practice, the results suggest that cognitively activating instruction, when implemented in ways that reduce anxiety and emphasize learning over evaluation, may support students’ emotional well-being and sense of belonging alongside academic development.

Several limitations should be noted. The cross-sectional nature of the data precludes causal inference, and all measures rely on student self-reports. Future research could employ longitudinal designs and incorporate observational measures of instruction to examine how changes in teaching practices influence students’ emotional security, social experiences, and belonging over time. In addition, extending this line of inquiry to other cultural contexts would further clarify the generalizability of the identified pathways.

Despite these limitations, the present study contributes to a growing body of research that positions classroom instruction as a key determinant of students’ psychological safety and social inclusion. By identifying emotional security as a robust and generalizable mechanism linking cognitively activating instruction to school belonging, the study advances understanding of how teaching practices shape students’ experiences beyond academic outcomes.

## Data Availability

Publicly available datasets were analyzed in this study. This data can be found here: the data used in this study are publicly available from the Organization for Economic Co-operation and Development (OECD). The Programme for International Student Assessment (PISA) 2018 dataset can be accessed through the OECD website (https://www.oecd.org/pisa/data/). All analyses in this study were conducted using anonymized secondary data, and no new data were generated or collected by the authors.

## References

[ref1] Allen K. A. Kern M. L. Vella-Brodrick D. Hattie J. Waters L. (2018). What schools need to know about fostering school belonging: a meta-analysis. Educ. Psychol. Rev. 30, 1–34. doi: 10.1007/s10648-016-9389-8

[ref2] Baumeister R. F. Leary M. R. (2007). “The need to belong: desire for interpersonal attachments as a fundamental human motivation,” in Interpersonal Development. 1st ed (New York, NY: Routledge), 33. 7777651

[ref3] Boman B. (2023). Is the SES and academic achievement relationship mediated by cognitive ability? Evidence from PISA 2018 using data from 77 countries. Front. Psychol. 14:1045568. doi: 10.3389/fpsyg.2023.1045568, 36910752 PMC9994354

[ref4] Breakspear S. (2012). The Policy Impact of PISA: An Exploration of the Normative Effects of International Benchmarking in School System Performance. OECD Education Working Papers No. 71. Paris: OECD Publishing.

[ref6] Dolton P. Marcenaro O. de Vries R. She P. W. (2018). Global teacher status index 2018. The Varkey Foundation. Available online at: https://hdl.handle.net/20.500.12799/6046 (Accessed March 01, 2026).

[ref7] Edwards D. (2020). PISA, Benessere e Sindacati Degli Insegnanti. Worlds of Education. Available online at: https://www.worldsofeducation.org/en/woe_homepage/woe_detail/16627/%E2%80%9Cpisa-well-being-and-teacherunions%E2%80%9D-by-david-edwards (Accessed March 07, 2026).

[ref8] Foley A. E. Herts J. B. Borgonovi F. Guerriero S. Levine S. C. Beilock S. L. (2017). The math anxiety-performance link: a global phenomenon. Curr. Dir. Psychol. Sci. 26, 52–58. doi: 10.1177/0963721416672463

[ref9] Goodenow C. Grady K. E. (1993). The relationship of school belonging and friends' values to academic motivation among urban adolescent students. J. Exp. Educ. 62, 60–71. doi: 10.1080/00220973.1993.9943831

[ref10] Govorova E. Benítez I. Muñiz J. (2020). Predicting student well-being: network analysis based on PISA 2018. Int. J. Environ. Res. Public Health 17:4014. doi: 10.3390/ijerph17114014, 32516891 PMC7312700

[ref11] Jia B. Zhu Z. (2025). Mathematical knowledge learning trajectories: an international comparative study. SAGE Open 15, 1–12. doi: 10.1177/21582440251375799

[ref12] Klieme E. Pauli C. Reusser K. (2009). “The Pythagoras study: investigating effects of teaching and learning in Swiss and German mathematics classrooms,” in The power of video Studies in Investigating Teaching and Learning in the Classroom, eds. Janík T. Seidel T. (Münster: Waxmann), 137–160.

[ref13] Lee J. (2009). Universals and specifics of math self-concept, math self-efficacy, and math anxiety across 41 PISA 2003 participating countries. Learn. Individ. Differ. 19, 355–365. doi: 10.1016/j.lindif.2008.10.009

[ref14] Li X. Kuo Y. L. Huggins T. J. (2024). Perceived feedback and school belonging: the mediating role of subjective well-being. Front. Psychol. 15:1450788. doi: 10.3389/fpsyg.2024.1450788, 39450132 PMC11499173

[ref15] Liu O. L. Wilson M. Paek I. (2008). A multidimensional Rasch analysis of gender differences in PISA mathematics. J. Appl. Meas. 9:18. 18180547

[ref16] Luttenberger S. Wimmer S. Paechter M. (2018). Spotlight on math anxiety. Psychol. Res. Behav. Manag. 11, 311–322. doi: 10.2147/PRBM.S141421, 30123014 PMC6087017

[ref17] Oden C. M. (2020). A Qualitative Exploration into the Decline of Japan's PISA Math and Science Test Scores and Japanese Students' Motivation to Learn Math and Science Skills: A Single Case Study. San Diego, CA, United States: Doctoral dissertation, Northcentral University.

[ref18] OECD (2013). PISA 2012 Results: Excellence through Equity: Giving every Student the Chance to Succeed, Vol. II. Paris: OECD Publishing.

[ref19] OECD (2017). PISA 2015 Results (Volume III): Students’ well-Being. Paris: OECD Publishing.

[ref20] OECD (2019). PISA 2018 Results (Volume III). Paris: OECD Publishing.

[ref21] Pekrun R. (2006). The control-value theory of achievement emotions: assumptions, corollaries, and implications for educational research and practice. Educ. Psychol. Rev. 18, 315–341. doi: 10.1007/s10648-006-9029-9

[ref22] Pekrun R. Goetz T. Titz W. Perry R. P. (2002). Academic emotions in students' self-regulated learning and achievement: a program of qualitative and quantitative research. Educ. Psychol. 37, 91–105. doi: 10.1207/S15326985EP3702_4

[ref23] Seidel T. Shavelson R. J. (2007). Teaching effectiveness research in the past decade: the role of theory and research design in disentangling meta-analysis results. Rev. Educ. Res. 77, 454–499. doi: 10.3102/0034654307310317

[ref24] Wong T. K. Parent A. M. Konishi C. (2019). Feeling connected: the roles of student-teacher relationships and sense of school belonging on future orientation. Int. J. Educ. Res. 94, 150–157. doi: 10.1016/j.ijer.2019.01.008

[ref25] Zhang D. Liu L. (2016). How does ICT use influence students' achievements in math and science over time? Evidence from PISA 2000 to 2012. Eurasia J. Math. Sci. Technol. Educ. 12, 2431–2449. doi: 10.12973/eurasia.2016.1297a

[ref26] Zhu Z. (2023). International comparative study of learning trajectories based on TIMSS 2019 G4 data on cognitive diagnostic models. Front. Psychol. 14:1241656. doi: 10.3389/fpsyg.2023.1241656, 37965657 PMC10641406

