# Supplementary material for: Cognitive activation and school belonging: the mediating roles of emotional security and social inclusion in East Asia
Source: Front Psychol. 2026 May 18;17:1795875. doi: 10.3389/fpsyg.2026.1795875 (PMC13222955; doi:10.3389/fpsyg.2026.1795875)
Supplement: Supplementary file 1 [file Table_1.docx]

**Appendix**

Table S1. Full Structural Regression Results Including Covariates

| **Outcome_label** | **Predictor_label** | **est** | **se** | **z** | **pvalue** | **std.all** |
| --- | --- | --- | --- | --- | --- | --- |
| Social inclusion | Cognitive activation | 0.032 | 0.005 | 5.972 | 0.000 | 0.060 |
| Social inclusion | Socioeconomic status (SES) | 0.009 | 0.005 | 1.820 | 0.069 | 0.016 |
| Social inclusion | Gender | -0.007 | 0.009 | -0.812 | 0.417 | -0.007 |
| Social inclusion | Immigrant background | 0.001 | 0.015 | 0.048 | 0.961 | 0.000 |
| Social inclusion | Grade repetition | 0.064 | 0.018 | 3.611 | 0.000 | 0.029 |
| Emotional security | Cognitive activation | 0.032 | 0.004 | 8.768 | 0.000 | 0.087 |
| Emotional security | Socioeconomic status (SES) | 0.001 | 0.004 | 0.229 | 0.819 | 0.002 |
| Emotional security | Gender | 0.002 | 0.006 | 0.376 | 0.707 | 0.003 |
| Emotional security | Immigrant background | 0.015 | 0.010 | 1.520 | 0.128 | 0.012 |
| Emotional security | Grade repetition | -0.053 | 0.013 | -3.920 | 0.000 | -0.035 |
| School belonging | Cognitive activation | 0.142 | 0.007 | 19.405 | 0.000 | 0.157 |
| School belonging | Social inclusion | 0.277 | 0.016 | 16.854 | 0.000 | 0.163 |
| School belonging | Emotional security | 0.621 | 0.023 | 27.441 | 0.000 | 0.253 |
| School belonging | Socioeconomic status (SES) | -0.002 | 0.007 | -0.273 | 0.785 | -0.002 |
| School belonging | Gender | -0.001 | 0.013 | -0.102 | 0.919 | -0.001 |
| School belonging | Immigrant background | 0.045 | 0.019 | 2.314 | 0.021 | 0.015 |
| School belonging | Grade repetition | -0.191 | 0.023 | -8.151 | 0.000 | -0.051 |
| Cognitive activation | Socioeconomic status (SES) | 0.012 | 0.009 | 1.280 | 0.201 | 0.011 |
| Cognitive activation | Gender | -0.019 | 0.015 | -1.296 | 0.195 | -0.009 |
| Cognitive activation | Immigrant background | 0.035 | 0.024 | 1.466 | 0.143 | 0.011 |
| Cognitive activation | Grade repetition | 0.191 | 0.045 | 4.205 | 0.000 | 0.046 |
